# The study design elements employed by researchers in preclinical animal experiments from two research domains and implications for automation of systematic reviews

**DOI:** 10.1371/journal.pone.0199441

**Published:** 2018-06-28

**Authors:** Annette M. O'Connor, Sarah C. Totton, Jonah N. Cullen, Mahmood Ramezani, Vijay Kalivarapu, Chaohui Yuan, Stephen B. Gilbert

**Affiliations:** 1 Department of Veterinary Diagnostic and Production Animal Medicine, College of Veterinary Medicine, Iowa State University, Ames, Iowa, United States of America; 2 Independent researcher, Guelph, ON, Canada; 3 Industrial and Manufacturing Systems Engineering, College of Engineering, Iowa State University, Ames, Iowa, United States of America; 4 Virtual Reality Applications Center, Iowa State University, Ames, Iowa, United States of America; 5 Department of Statistics, Iowa State University, Ames, Iowa, United States of America; Johns Hopkins University Bloomberg School of Public Health, UNITED STATES

## Abstract

Systematic reviews are increasingly using data from preclinical animal experiments in evidence networks. Further, there are ever-increasing efforts to automate aspects of the systematic review process. When assessing systematic bias and unit-of-analysis errors in preclinical experiments, it is critical to understand the study design elements employed by investigators. Such information can also inform prioritization of automation efforts that allow the identification of the most common issues. The aim of this study was to identify the design elements used by investigators in preclinical research in order to inform unique aspects of assessment of bias and error in preclinical research. Using 100 preclinical experiments each related to brain trauma and toxicology, we assessed design elements described by the investigators. We evaluated Methods and Materials sections of reports for descriptions of the following design elements: 1) use of comparison group, 2) unit of allocation of the interventions to study units, 3) arrangement of factors, 4) method of factor allocation to study units, 5) concealment of the factors during allocation and outcome assessment, 6) independence of study units, and 7) nature of factors. Many investigators reported using design elements that suggested the potential for unit-of-analysis errors, i.e., descriptions of repeated measurements of the outcome (94/200) and descriptions of potential for pseudo-replication (99/200). Use of complex factor arrangements was common, with 112 experiments using some form of factorial design (complete, incomplete or split-plot-like). In the toxicology dataset, 20 of the 100 experiments appeared to use a split-plot-like design, although no investigators used this term. The common use of repeated measures and factorial designs means understanding bias and error in preclinical experimental design might require greater expertise than simple parallel designs. Similarly, use of complex factor arrangements creates novel challenges for accurate automation of data extraction and bias and error assessment in preclinical experiments.

## Introduction

### Rationale

Systematic reviews are increasingly incorporating data from preclinical animal experiments [1–5]. Accurate and efficient interpretation of the study design used in such experiments is an important component of that process, because a unique aspect of systematic reviews is the assessment of bias and errors in the study design, in addition to extraction of the effect sizes and effect size precision. Here we refer to "study design" as the procedural outline for conducting an investigation. Therefore, a study design is comprised of multiple "design elements," which include use (or not) of randomization, use (or not) of blinding, how often the outcome is measured, the type of control group used, and how the experimental factors are arranged [6]. To assess bias and errors and extract the study results, it is critical that the reviewers understand the study design and know which elements are reported. For a systematic reviewer, a study described as an "*individually randomized*, *3 by 2 factorial design blocked by sex*, *with repeated measures and blinded outcome assessment*" immediately reveals the design element options employed by the investigators. It also conveys that the investigators used design element options that relate to risk of systematic biases (randomized and blinded) and that have the potential to create unit-of-analysis errors (repeated measures). A unit-of-analysis error occurs when the unit of allocation of the intervention is different from the unit used in the statistical analysis. Further, this description of the study ensures that the reviewer knows the results will likely contain an assessment of two main effects and an interaction (factorial design).

Assessment of the study design is a very labor- and time-intensive process, as it requires considerable time and expertise to recognize specific design elements such as split-plot designs. Automated recognition of design elements would considerably speed up this aspect of systematic reviews. However, effective systematic review automation might requires knowledge of which design elements are commonly employed, as such information will enable prioritization of targets for automation efforts.

Although many studies have described the frequency with which randomization and blinding are reported by investigators in preclinical experiments [7], our focus was to extend to the description of less commonly assessed design elements, particularly those that relate to replicates and the arrangement of study factors. Our rationale for selecting this focus is that these elements are under studied yet important design elements that impact study validity and accurate extraction of study results [8–12].

### Objective

Our long-term goal is to develop automated tools for the recognition of design elements in research publications, as recognition of important study design elements requires considerable expertise, and automated classification of design elements will enable more accurate, rapid, and cost-effective risk-of-bias and error assessment and extraction of study results. Working towards that longer-term goal, the objective of this study was to identify and assess the frequency of design elements in preclinical animal experiments. Such information will be needed so that automation methods can focus on identifying the most commonly employed design elements and therefore maximize value to reviewers.

## Materials and methods

This study is an observational survey using manuscripts describing preclinical animal experiments from systematic reviews in two broad topic areas: brain trauma/stroke and toxicology.

### Data sources

Manuscripts included described primary research of a single comparative animal experiment (published in English). Only *in vivo* studies were eligible. If an eligible manuscript also contained an *in vitro* or *ex vivo* intervention element, the manuscript as a whole was excluded. The single-study criterion was necessary for a companion project using the same set of studies. The datasets for each topic area contained 100 manuscripts. One dataset was obtained from the CAMARADES (Collaborative Approach to Meta-Analysis and Review of Animal Data from Experimental Studies) group and described animal models of stroke/brain trauma. The second dataset was obtained from the citation lists of four systematic reviews that evaluated animal models for toxicology. Further details of how the corpus was obtained are provided in S1 Text.

### Eligible studies

Initial screening of manuscripts for the corpus was conducted using the online systematic review software DistillerSR® (Ottawa, ON, Canada, https://www.evidencepartners.com/). Initial eligibility assessment was performed based on the abstract, keywords, introduction, and the materials and methods sections. Studies were eligible for assessment if published in English (the full text, not just the abstract), if they were primary research of a comparative intervention or assessment of brain trauma/stroke outcomes in non-primate mammals, consisting of only one experiment, and assessing only interventions applied to the whole animal (i.e., no *in vitro* or *ex vivo* level interventions).

Two independent reviewers (JC and ST) with backgrounds in study design pilot-tested the initial screening (eligibility) form on 30 studies. Subsequent to the pilot-testing, only one reviewer (JC or ST) was required to determine study eligibility.

After eligibility assessment, 100 references, out of the 213 eligible studies in the CAMARADES dataset, were selected using a random number sequence generator (https://www.random.org/sequences). The rationale for the sample size of 100 studies was to enable 95% confidence of the ability to identify design elements present in at least 5% of manuscripts assuming 100% sensitivity and 100% specificity of detection (http://epitools.ausvet.com.au/content.php?page=Freedom), which in the absence of prior data seemed a pragmatic goal for detection of design elements. It was decided by the 1^st^ author that if a design element occurred in fewer than 5% of the papers, then it was rare enough to ignore for this report. To extract the data, a PDF annotation tool (AFLEX interface) was developed which enabled pre-specified design elements to be tagged/associated with specific text within the full-text PDF [13]. This web-based tool allows the user to upload a PDF, highlight passages of text in them, and tag those passages with the design elements. E.g., a user might select a sentence that provides evidence for the unit of analysis being the group and the arrangement of factors being parallel. That highlighted sentence can then be tagged with Group and Parallel. After tagging, the tool allows easy review of the evidentiary sentences for that article, or a review of all Group sentences across all tagged articles, etc. Currently the tool is being used internally by the authors, but it could become available for public use.

The design element assessment extraction form was pilot-tested by two independent reviewers (JC and ST). After pilot-testing, each study was assessed and extracted by the two independent reviewers (JC and ST). To identify and resolve conflicts about design elements and supporting text, an RStudio-based Shiny [14, 15] web interface was developed, which identified where design elements and text were not the same for both reviewers. Following conflict resolution or adjudication by a third reviewer (AOC), any necessary changes to the final dataset were made.

### Identification of design elements used and supporting text collection process

The design elements sought were selected based on previous experience with identifying and extracting study design elements and consisted of a comprehensive suite of elements relevant to comparative preclinical animal experiments. As part of assessing whether the list was comprehensive, several risk-of-bias tools proposed for animal experiments were reviewed to determine which design elements would relate to systematic bias and unit-of-analysis errors [7, 16, 17].

The selected design elements are: 1) comparison group, 2) unit of allocation of the interventions to study units, 3) arrangement of factors, 4) method of factor allocation to study units, 5) concealment of the factors during allocation and outcome assessment, 6) independence of study units, and 7) nature of factors. For each design element, there are options that investigators might employ. For example, for the design element "arrangement of factors" investigators can choose from a parallel arrangement of factors, a single-level factorial arrangement, a split-plot-like factorial arrangement, or a cross-over arrangement. The suite of design elements and options are described in Table 1. The suite of design elements and their associated validity and bias domains can be seen in S1 Table.

**Table 1:** Design element groups and annotation options for use with an AFLEX (automatic functional language recognition/EXtraction) interface for annotating portable document format files.

Design element	Element options	Comments
**Comparison group**	None	There is only one group in the study and this group received the intervention. This group may serve as its own control, i.e., the outcome is assessed prior to and following application of the intervention(s).
	Concurrent	The design has two or more comparison groups that occur at the same time.
	Historic	The design has at least one comparison group that completed the study before the other comparison group(s) entered the study.
**Unit of concern**	Group	The factors are applied at the level of the group, such as cage or other housing.
	Individual	The factors are applied at the level of the individual.
	Nested	There are two or more hierarchical levels of the factors (e.g., one factor applied to pregnant mother, and a second factor applied to the pups).
**Arrangement of factors**	Parallel	Two or more experimental groups are followed over time. Interaction between factors is not studied.
	Cross-over	At least two experimental groups are in the study, and the groups swap interventions.
	Complete factorial	At least two factors are studied and all possible combinations of these factors are present in the design. These factors are applied at the same level. (all factors applied at single level).
	Incomplete factorial	At least two factors are studied but not all possible combinations of these factors are present in the design. These factors are applied at the same level. (all factors applied at single level).
	Split-plot	Factors are investigated at two or more hierarchical levels in the study, i.e., one or more factors are nested within another factor (e.g., whole mouse, two or more tissues within the mouse).
**Allocation**	Random	Refers to the use of a random allocation methods
	Systematic	Refers to the use of alternation methods.
	Minimization	Minimization includes matching on known confounders based on previously enrolled animals.
	Haphazard	A method that is none of the above, such as allocating the next intervention to the next mouse caught. Rarely is the word "haphazard" used; however, a described method might appear haphazard.
**Concealment**	Blinded intervention allocation	The investigators indicated whether the allocation sequence was concealed prior to enrolment.
	Blinded outcome assessment	The investigators indicated whether the outcome assessor(s) was/were blinded to the intervention groups.
**Independence**	Pseudo-replication	Pseudo-replication is considered multiple measures of an outcome designed to capture random experimental noise, i.e., multiple pups within a litter when the dam had been allocated to treatment or multiple tissue sections within an animal.
	Repeated measures	Repeated measures refers to multiple measurements of an outcome when a factor is varied. The multiple outcome measurements are spread across a factor of potential interest, such as time or decibels.
**Investigator-identified study design**	Investigator-Identified Study Design	The study design, as identified by the study investigator(s) in the Title, Abstract, Keywords, Objectives, and/or Methods sections of the article.
**Nature of the factors allocated**	All could be randomized Some could be randomized None could be randomized	The investigators examined only factors that could be randomized (e.g., drugs, exercise treatments, diets, etc.) The investigators examined a mixture of factors that could be randomized and factors that could not be randomized. The investigators examined only factors that could not be randomized (e.g., sex, genotype, age, and tissue type (when more than 1 type of tissue was sampled per experimental unit)).

The methods section of each manuscript was searched for text that indicated the design elements described by the investigators. If identified, the option employed by the investigators and a text description of the option were extracted using *de novo* software (Table 1). In addition, text in the title, abstract, introduction, and the materials and methods section were surveyed for any investigator-identified study design label and, if present, this information was extracted.

Certain design elements must be present in an experimental study; for example, all studies must identify a unit of allocation, an arrangement of factors, and a method of allocation of factors to study units. When it was not possible to discern the options used based on the investigators' description, these design elements were referred to as "unclear". Other design elements are optional, such as concealment of factors during allocation or outcome assessment, repeated measures, or the use of pseudo-replication. If no text was found to describe these elements, this was coded as having "no discernable description (NDD)" for that design element.

To ensure a consistent approach to element and option recognition, the following standards were employed:

- In order to determine whether a control group was concurrent, text was selected that described the division or allocation of the study population into groups.
- In order to determine whether the unit of allocation was at the individual level, we required the investigators to provide either a dosage (e.g., mg/kg) or a route of administration (e.g., intravenous, intraperitoneal) that could only be delivered individually. Simply providing a concentration of the intervention in the water or food was not sufficient for the reviewers to determine the unit of allocation, unless the authors also explicitly described the housing as individual.
- We differentiated language that suggested pseudo-replication from language that suggested repeated measurement of outcomes, although these approaches both refer to replicates [9]. *Pseudo-replication* refers to multiple measures of an outcome designed to capture random experimental noise, i.e., multiple pups within a litter when the dam had been allocated to treatment or multiple tissue sections within an animal. *Repeated measurement* refers to multiple outcome measurements when a factor of interest varies, such as time or decibel level. Descriptions of measures that were unlikely to be related to the outcome were not extracted, as such information did not relate to the extracted results. For example, repeatedly measuring body temperature while the animal was under anesthesia was to ensure animal health and was therefore unlikely to be reported in the results. Two approaches to recognition of repeated measures were used: 1) if the investigators described a process of repeated measurements of outcomes on a study unit, and 2) if the statistical methods described an approach to control for repeated measures, such as "a repeated measures ANOVA was conducted".
- For the arrangement of factors, when the factors were assigned to the same level of animal and the interaction between multiple factors was of interest, this was considered a *single-level factorial design*. A factorial design was considered complete when every possible combination of factors was represented by an arm of the design [11].

A common feature of preclinical studies is a "sham" arm, which is often included for the purposes of quality control. This "sham" arm is often paired with a factorial design, and as a consequence, could be mistaken for part of an incomplete factorial design. The difference between these designs is based on the nature of the "single" arm. A sham arm consists of animals that received neither an intervention, nor a challenge (where "challenge" was induction of stroke in the CAMARADES dataset). The sham arm is a quality control feature of the study, rather than having an outcome that is truly of interest. Data from animals in the sham arm may function as a baseline for the outcomes from control groups (which received the challenge) and treatment groups (which received both the challenge and an intervention.

A *split-plot-like arrangement* referred to a factorial arrangement where one factor is nested within the other.

If the arrangement of factors could not be deciphered based on the investigators' text, the portion of the text describing the overall organization of the factors was extracted and labeled "unclear" as the design element.

To describe the findings, we calculated the frequencies of design elements and options for the selected studies.

## Results

### Study characteristics

#### Investigator-identified study design

No investigator reported a specific study design name such as "2 by 2 factorial" in any of the 100 studies extracted from the CAMARADES (stroke/brain trauma) dataset. Only seven studies from the toxicology dataset contained an investigator-identified study design. All seven of these studies were described by the investigators as factorial designs. Interestingly, two of these seven studies appeared to be split-plot-like designs based on the investigators' description of the arrangement of factors. Of course, split-plot is a unique sub-group of factorial design; therefore, the description of these studies as factorial is technically correct. However, the use of the term "split-plot-like design" is preferable, as it would alert reviewers more quickly to the potential for unit-of-analysis errors in the manuscripts.

#### Frequency of study design elements and options

Table 2 shows the frequency of reporting of design elements in the two datasets.

**Table 2:** Frequency of description of design elements and design element options in the two preclinical datasets evaluated (CAMARADES (brain trauma/stroke) and toxicology).

Design element	Element options	CAMARADES N = 100	Toxicology N = 100
Control group	None	0	0
	Concurrent	98	92
	Historic	0	0
	Unclear	2	8
Unit of concern	Group	0	12
	Individual	92	37
	Nested	0	17
	Unclear	8	34
Arrangement of the factors	Complete factorial	27	42
	Cross-over	0	0
	Incomplete factorial	12	11
	Parallel	58	24
	Split-Plot	0	20
	Unclear	3	3
Allocation	Haphazard	0	0
	Minimization	0	0
	Random	79	62
	Systematic	0	0
	Unclear	21	38
Concealment (a)	Intervention allocation	12	0
	NDD*	88	100
Concealment (b)	Outcome assessment	60	12
	NDD*	40	88
Independence (a)	Pseudo-replication	44	50
	NDD*	56	50
Independence (b)	Repeated measures	40	59
	NDD*	60	41
Investigator-identified study design	Provided	0	7
	NDD*	100	93
Nature of the factors	All could be randomized	94	69
	Some could be randomized	5	30
	None could be randomized	0	0
	Unclear	1	1

* NDD = no discernable description: neither reviewer/reader was able to find text that described this element.

One of the most important findings is that, despite the absence of specific design labels, the reviewers were almost always able to confidently determine the arrangement of factors used by the investigators. This means that this information about the design element is not missing, as is often the case for other important design elements such as randomization or blinding. Authors appear to not routinely use regular expressions such as "2 by 2-factorial design" or "split-plot design" and instead describe these elements using more complex language forms than might be expected.

Another important finding is that more variation in the unit of allocation was observed in the toxicology dataset than in the brain trauma/stroke dataset. The toxicology dataset included more nested, group, and unclear allocations. The factors studied in our particular toxicology dataset tended to be those conducive to application to the food or water and if animals were group-housed, it was probably more expedient for the investigators to allocate these factors at the group level by adding them to the food or water of group-caged animals. In the brain trauma/stroke dataset, the interventions of interest were usually those that could only be applied at the individual level (e.g. injectable drugs) and cross-generational effects of the intervention were not of interest to the investigators. By contrast, investigators in our toxicology dataset studies were often interested in cross-generational effects of the toxins of interest, hence we found that the factors were often applied to pregnant dams and their offspring (nested allocation).

Similarly, more variation was observed in the arrangement of factors in the toxicology studies compared to the brain trauma/stroke studies. Important for unit-of-analysis errors, 20% of the toxicology studies used language that suggested a split-plot-like arrangement of factors of interest, although as previously noted, no investigator used the term "split-plot". As with group-level allocation, the use of a split-plot-like arrangement of factors (one or more subplots nested within a whole plot) suggests that unit-of-analysis errors could occur. Reviewers would benefit from being alerted to this potential, as it enables them to verify that the study correctly adjusted for the whole-plot error term in the statistical analysis [12].

With respect to allocation to treatment group, not surprisingly, randomization was the only reported method of allocation. The studies not indicating randomization did not report which method was used to allocate the interventions to the study units. Similarly, blinding of allocation and outcome assessment were rarely described in preclinical studies.

Language that suggests the potential for unit-of-analysis concerns as a result of pseudo-replication and repeated measures was common in both datasets. Almost 50% of studies used language that described pseudo-replication and/or repeated measures [8, 9]. Our goal with this study was not to determine whether the investigators addressed these concerns when conducting their analysis. However, it is relevant to note that sometimes, though not always, the investigators' description of the element also indicates that the unit-of-analysis errors concern was addressed. This has implications for efficient text extraction and bias or error assessment. For example, in the toxicology dataset, 53 manuscripts used language that suggested repeated measures, such as in the following text:

"Offspring were weighed at 7 day intervals and food intake over 24 hours was measured at 25 day intervals."[18]

However, only 26 of those 53 studies also provided language in the methods and materials section that suggested that this unit-of-analysis concern had been addressed. For example:

"The repeated measures ANOVA was used for the acquisition phase of the MWM and rMWM (with the repeated measure: trial block), followed by a Bonferroni post hoc to analyze possible interactions between trial block, genotype and/or diet." (emphasis added) [19]

In the CAMARADES (brain trauma/stroke) dataset, 28 of the 40 studies that used language suggesting repeated measures did not also include language that indicated this had been addressed in the statistical analysis. Similar results were found for pseudo-replication; for the toxicology dataset, 46 studies used language that suggested pseudo-replication, but 34 (74%) of these studies did not clearly indicate how this was addressed analytically. For the CAMARADES dataset, in 29 of 44 (66%) studies the investigators' description of pseudo-replication did not also contain evidence of the solution. An example of language the reviewers considered to suggest the issue and the resolution is:

"The digital reading (in Newtons) of three successive trials were obtained for each mouse, averaged and used for data analysis." [20]

Also of interest was the finding that many studies, especially in the toxicology dataset, included factors of interest that could not be randomized. This was most seen for factors related to factors of genotype or sex, for example:

"In order to determine the contribution of both genetic TXNIP-deletion (TKO) and the pharmacologic TXNIP inhibition with RES on outcome/recover after embolic middle cerebral artery occlusion (eMCAO) stroke, the total 64 mice (WT and TKO) were separated into following groups: WT mice subjected to sham operated control + vehicle treatment group I (sham only); WT mice subjected to eMCAO + vehicle treatment group II (WTeMCAO only); WT mice subjected to eMCAO + RES (5mg/kg) treatment group III (WTeMCAO + RES only) and TKO mice subjected to eMCAO group + vehicle treatment IV (TKO-eMCAO only)." [20]

Our interpretation of this design is that genotype was a factor of interest, but animals could not be randomized to genotype in the true sense. This has implications for automated risk-of-bias assessment, as it is not possible to assume that all factors studied in preclinical experiments can be randomized to group.

## Discussion

The data suggest that investigators report the use of a variety of design elements in preclinical studies. To date, much of the focus on comprehensive reporting in biomedical research has been on the design elements that relate to selection bias and detection bias. The design element "allocation to group" is related to selection bias, and incorporation of blinded outcome assessment relates to detection bias [1, 6, 17, 21–25]. This focus is likely a function of three factors. First, in the literature on human studies the reporting of these design elements has been evaluated for years and continues to be the focus of many studies; second, there is empirical evidence of an association between reporting of these elements and the effect size of intervention studies [26–31]. Finally, the assessment of these factors does not require advanced understanding of study design because authors use typical expressions or keywords more commonly to describe the options for these design elements, i.e., randomization and blinding, and therefore the task of assessment of reporting is relatively simple.

Less focus has been applied to the reporting of elements that may impact the potential for unit-of-analysis errors. Interestingly, our data suggest that such elements are actually quite common in preclinical studies. For example, in the two datasets we evaluated, almost 50% of investigators opted to include a design element that suggested the potential for repeated measures or pseudo-replication, and 20% of the studies in the toxicology dataset described split-plot-like designs. Regrettably, we could not identify other reviews that evaluated design elements associated with potential unit-of-analysis errors in other sets of preclinical studies or human studies. One report of preclinical researchers did study investigator awareness of bias and error avoiding design elements and included the option of independent observations. Surprisingly, many investigators identified independent observations as an approach to avoiding attrition bias (~40%), performance bias (~50%), selective reporting (~30%), detection bias (~50%), publication bias (~35%), and selection bias (~38%) [32]. While independent observations are important, they are not related to any of these sources of bias. The survey did not ask questions about avoiding unit-of-analysis errors.

The findings also illustrated the complexity of designs that include multiple elements. For example, some reviewers might assume that all split-plot designs use a nested allocation; however, this is not the case, for several reasons. To illustrate, the text below describes a split-plot design with allocation of the diet to dams (whole plot) and then the sex of the pup is identified as a sub-plot factor.

"Once bred, pregnant dams (n = 6/group) were fed one of four diets; (1) control diet, (2) high fat (HF) diet, (3) control + methyl donor supplementation (Control + Met) and (4) high fat + methyl donor supplementation (HF + Met). …One animal per litter was used in individual experiments, to control for any litter effect. …Male and female offspring were followed longitudinally and tested at the following time points (1) 12 and 20 weeks of age-metabolic assessments, (2) 40 weeks of age- fat and sucrose preference test, and (3) 50 weeks of age-brain collection for gene expression and methylation assays." [33]

To understand the potential sources of bias and error in this design, substantial knowledge about the design and thorough interpretation of information is needed. First, the investigator cannot randomize the sub-plot factor (sex) as it is a characteristic of the animal. Therefore, the "nested" allocation, which may be considered the default for a split-plot design, is not appropriate in this study. Only the whole-plot factor (diet) can be "allocated" to the dam. Therefore, it is only relevant to assess the risk of bias due to allocation at the whole plot not the sub-plot level because the nature of the factor (sex) means it cannot be randomized. Further, diet is a factor that could be allocated at the individual or group level, and the investigators did not specify the unit of allocation. As a result, the description above might suggest the potential for pseudo-replication at the whole plot level if all the dams from one group where housed in the same cage and this correlation was not addressed in the design. This example illustrates why it is necessary to evaluate all design elements to fully understand the potential for systematic bias or unit-of-analysis errors estimation in preclinical studies.

We also found that studies in the two datasets commonly used complex arrangements of factors. In the CAMARADES (brain trauma/stroke) dataset, 40% of the 100 studies utilized some form of factorial design, and in the toxicology dataset, more than 75% of the 100 studies used some form of factorial design. Further, 25% of the studies in the toxicology dataset where spit-plot-like. Given that factorial designs often have interactions between main effects, reviewers and automated methods extracting data from preclinical studies will need to understand how to appropriately extract effect sizes and variance estimates from results with and without significant interactions. As a first step to assessing unit-of-analysis errors, reviewers and automated methods would need to be able to recognize a split-plot design so that the validity of the approach to analysis could be assessed. We have not previously seen the frequency of factor arrangement types assessed in preclinical animal experiments or human clinical trials. Our impression is that parallel and cross-over designs may predominate in human studies. For example, a search of trial titles for intervention studies submitted to Clinical Trials.Gov (https://clinicaltrials.gov) identified only 142 studies that used "factorial" in the title, yet 8146 titles included "parallel" in the title and 6100 used the term "cross-over".

A limitation of this study is that it is based on only two topic areas of preclinical studies with a relatively small subsample of 200 studies. The reason for this limited number relates to resources, i.e., it takes considerable expertise and time to identify all important design elements in a manuscript. This limitation re-enforces our original motivation–that design elements beyond randomization and blinding are also important for understanding study design and currently few authors clearly provide this information.

We would propose that three groups could use the findings here. Although authors do write about the design in a manner than enables experienced researchers to recognize the design elements, authors could better help others understand the design by using more key-terms for design elements. For example, describing a design as a 2 by 2 factorial design or that it contains a repeated measure elements improves the translation of research finings to end users. This does however require that authors are explicitly aware of the design elements employed and the appropriate terminology. Peer-reviews and editors could also encourage the use of common key-terms for design elements. For end users, in particular systematic reviewers, the information provided suggests that they should not currently rely upon authors to use key-terms to identify design elements, especially those with the potential to impact unit-of-analysis errors. Instead systematic reviewers in pre-clinical health should be aware that the features can be common, and should be considered when seek to extract valid estimates of effect size and precisions for use in systematic reviews.

## Conclusions

This study documents that investigators of primary research in preclinical animal experiments employ many design elements. We find it particularly interesting that many of these design elements could relate to unit-of-analysis errors (nested allocation, group allocation, split-plot-like designs, pseudo-replication, and repeated measures). However, the potential for unit-of-analysis error is rarely discussed or included in risk-of-bias assessments in preclinical animal experiments in systematic reviews. It is rare for investigators in this area of research to specifically name the study design used. Reporting of allocation concealment is also rare. The toxicology dataset described more nested, group, and unclear allocations, indicating that reviewers in this topic area need to be particularly careful when reading these studies to understand whether unit-of-analysis errors suggested by the design are properly addressed in the statistical analysis.

## Supporting information

S1 Text (DOCX)

S1 Table (DOCX)
