# Supplementary material for: The study design elements employed by researchers in preclinical animal experiments from two research domains and implications for automation of systematic reviews
Source: PLoS One. 2018 Jun 28;13(6):e0199441. doi: 10.1371/journal.pone.0199441 (PMC6023607; doi:10.1371/journal.pone.0199441)
Supplement: S1 Text — (DOCX) [file pone.0199441.s001.docx]

**Study population sources**

Eligible manuscripts were selected from two topic areas that were chosen for pragmatic reasons: ready access and expected variation. The first group of studies came from The Collaborative Approach to Meta-Analysis and Review of Animal Data from Experimental Studies (CAMARADES) group at the University of Edinburgh. The CAMARADES group has expertise in systematic reviews and meta-analyses in animal studies and, in particular, traumatic brain injury (e.g., stroke) and neurological disease. CAMARADES provided a citation dataset of 500 preclinical trials previously included in systematic reviews of brain trauma/stroke. These 500 references (out of 1152 publications, all of which had been screened for MCAO (stroke model)), were randomly selected using the random number generator [9].

The second group of studies was selected from systematic reviews that had been conducted in the field of environmental toxicology. We selected 80 eligible studies from the three most recently conducted reviews listed on the National Toxicology Program website (https://ntp.niehs.nih.gov/pubhealth/hat/noms/index.html) (fluoride, folic acid, and perfluorooctanoic acid (PFOA)/perfluorooctane sulfonate (PFOS)) at the start of the project (Dec 2016) that included animal studies [33-35] [30-32]. Because these reviews provided an insufficient number of studies, we also selected studies from an EFSA (European Food Safety Authority) mercury review, purposefully chosen because it was recent, included animal studies, and related to toxicology [36][33].

**Study selection**

Of the 500 CAMRADES references screened for eligibility, 26 references were excluded because the full text was not available in English (25 were in Chinese, 1 was in Russian), 2 were not primary research, 121 described *in vitro* and/or *ex vivo* experiments, and 51 did not assess an intervention. Of the remaining 300 references (all of which were *in vivo* primary research animal intervention studies in English), in 8 studies the number of experiments conducted was unclear, and another 89 references described more than one experiment, leaving 203 references eligible for evaluation.

During data extraction, an additional 6 studies had to be excluded because the corresponding Portable Document Format files could not be annotated within the AFLEX interface (the tool used to extract the data) because of problems with the files (file size too big, document was a scanned photocopy so that text could not be highlighted). In each case, the study was excluded and the next eligible study, as determined by the random number sequence generator, was selected for data extraction.
