# Supplementary material for: The study design elements employed by researchers in preclinical animal experiments from two research domains and implications for automation of systematic reviews
Source: PLoS One. 2018 Jun 28;13(6):e0199441. doi: 10.1371/journal.pone.0199441 (PMC6023607; doi:10.1371/journal.pone.0199441)
Supplement: S1 Table — (DOCX) [file pone.0199441.s002.docx]

**S1 Table.**

| **Design element** | **Bias or validity of domain** |
| --- | --- |
| Comparison group | Inclusion of a comparison group serves to enable comparison and therefore estimation of the effect. |
|  |  |
|  |  |
| Unit of concern | Knowledge of the unit of concern is critical for understanding if the estimate of the variance is correct. Most statistical tests assume independence of study units. If this assumption is violated, the estimation of the variance will be incorrect and this will increase the potential for a type 1 error or result in an overly precise estimate of the effect size. |
|  |  |
|  |  |
| Arrangement of factors | Knowledge of the arrangement of the factors contributes to understanding if the estimate of the variance is correct. Cross-over designs and nested designs create structures where observations are not independent and therefore the estimation of variance can be incorrect if this is ignored and increase the potential for a type 1 error or result in an overly precise estimate of the effect size. |
| Allocation | The approach to allocation is important for controlling confounding. Use of non-random methods such as quasi-random systematic allocation or haphazard allocation have been associated with bias. |
| Concealment | Concealment of the intervention allocation is designed to reduce selection bias when employed at enrolment, and to reduce information bias when employed at outcome measurement. |
| Independence | Correct estimation of variance, and therefore precision and correct hypothesis testing, requires correct identification of the independent unit of study. |
| Nature of the factors allocated | As failure to randomize is considered an important element, it was important to verify that all studies had at least one factor of interest that could be randomized. |
